# Supplementary material for: The role of grain boundary scattering in reducing the thermal conductivity of polycrystalline XNiSn (X = Hf, Zr, Ti) half-Heusler alloys
Source: Sci Rep. 2017 Oct 23;7:13760. doi: 10.1038/s41598-017-14013-8 (PMC5653807; doi:10.1038/s41598-017-14013-8)
Supplement: Supplementary file 1 — Supplementary information [file 41598_2017_14013_MOESM1_ESM.pdf]

Supporting information

The role of grain boundary scattering in reducing the thermal conductivity of polycrystalline *X*NiSn (*X* = Hf, Zr, Ti) half-Heusler alloys

Matthias Schrade[1], Kristian Berland[1], Simen N. H. Eliassen[1,2], Matylda N. Guzik[1,3], Cristina Echevarria-Bonet[3], Magnus H. Sørby[3], Petra Jenuš[4], Bjørn C. Hauback[3], Raluca Tofan[1], Anette E. Gunnæs[1], Clas Persson[1], Ole Martin Løvvik[1,5], and Terje G. Finstad[1]

[1] Centre for Materials Science and Nanotechnology, Department of Physics, University of Oslo, Gaustadalléen 21, NO-0349 Oslo, Norway

[2]Department of Materials Science and Engineering, Norwegian University of Science and Technology, Norway

[3] Physics Department, Institute for Energy Technology, NO-2007 Kjeller, Norway

[4] Jožef Stefan Institute, Department for Nanostructured Materials, Ljubljana, Slovenia

[5] SINTEF Materials and Chemistry, Forskningsveien 1, NO-0314 Oslo, Norway

S.1. SR-PXD refinement results

1. **TiNiSn**

Phases identified from Rietveld analysis:

- **TiNiSn**: 95.8(4) % of total sample mass, crystallite size: d = 64 nm; microstrain : η = 0.05(1)% (R_{Bragg} = 5.08, R_F = 2.52)
- **$TiNi_2Sn$**: 1.0(1) % of total sample mass (R_{Bragg} = 16.9, R_F = 10.5)
- **Sn** 3.2(1) % of total sample mass (R_{Bragg} = 6.79, R_F = 6.42)

R_p = 6.93, R_{wp} = 9.68, R_{exp} = 0.01

2. **ZrNiSn**

Phases identified from Rietveld analysis:

- **ZrNiSn**: 100 % of total sample mass, crystallite size: d = 96 nm; microstrain : η = 0.08(1)%

R_p = 9.21, R_{wp} =11.8, R_{exp} = 0.01, R_{Bragg} = 8.96, R_F = 4.20

Though no other phase was included in the refinement there are peaks suggesting the presence of **Sn** but they account for not more than 1-2 % of total sample mass.

3. **HfNiSn**

Phases identified from Rietveld analysis:

- **HfNiSn**: 91.4(6) % of total sample mass, crystallite size: d = 49 nm; microstrain : η = 0.008(3)% (R_{Bragg} = 16.9, R_F = 10.5)
- **$HfNi_2Sn$**: 0.04(2) % of total sample mass (R_{Bragg} = 29.2, R_F = 23.8)
- **Hf**: 7.9(3) % of total sample mass (R_{Bragg} = 20.5, R_F = 11.1)
- **Sn**: 0.7(3) % of total sample mass (R_{Bragg} = 37.7, R_F = 31.1)

R_p = 5.24, R_{wp} = 7.67, R_{exp} = 0.02

4. **(Ti,Zr)NiSn**

Phases identified from Rietveld analysis:
5 Half-Heusler phases:

- **$Ti_{0.30}Zr_{0.70}NiSn$**: 32.8(6) % of total sample mass (R_{Bragg} = 6.27, R_F = 3.55)
- **$Ti_{0.82}Zr_{0.18}NiSn$**: 10.4(2) % of total sample mass (R_{Bragg} = 10.2, R_F = 7.61)
- **$Ti_{0.10}Zr_{0.90}NiSn$**: 13.2(4) % of total sample mass (R_{Bragg} = 8.76, R_F = 5.89)
- **$Ti_{0.58}Zr_{0.42}NiSn$**: 35.6(4) % of total sample mass (R_{Bragg} = 6.72, R_F = 4.04)
- **$Ti_{0.33}Zr_{0.67}NiSn$**: 8.1(1) % of total sample mass (R_{Bragg} = 6.93, R_F = 4.31)

And other minor impurities including **Sn**, not included in the analysis. Crystallite sizes *d* vary in the range of 20-100 nm among identified phases.

R_p = 6.29, R_{wp} = 7.80, R_{exp} = 0.01

5. **(Ti,Hf)NiSn**

Phases identified from Rietveld analysis:

2 Half-Heusler phases:

- **$Ti_{0.31}Hf_{0.69}NiSn$**: 57.8(5) % of total sample mass, crystallite size: d = 56(3) nm; microstrain: η = 0.39(5) % (R_{Bragg} = 6.79, R_F = 5.01)
- **$Ti_{0.69}Hf_{0.31}NiSn$**: 42.2(6) % of total sample mass, crystallite size: d = 135(7) nm; microstrain: η = 0.21(4) % (R_{Bragg} = 8.62, R_F = 6.42)

and other minor impurities including **Sn**, not included in the analysis.

R_p = 5.37, R_{wp} = 8.25, R_{exp} = 0.02

6. **(Zr,Hf)NiSn**

Phases identified from Rietveld analysis:

- **$Zr_{0.62}Hf_{0.38}NiSn$**: 100 % of total sample mass, d = 79(1) nm; microstrain: η = 0.12(1)%

R_p = 4.70, R_{wp} =5.51, R_{exp} = 0.05, R_{Bragg} = 9.02, R_F = 5.18

7. **(Ti, Zr,Hf)NiSn**

Phases identified from Rietveld analysis:

- **$Ti_{0.40}Hf_{0.30}Zr_{0.30}NiSn$**: 37(1) % of total sample mass, crystallite size: d = 58(3) nm; microstrain: η = 0.24(4) % (R_{Bragg} = 7.92, R_F = 6.00)
- **$Ti_{0.60}Hf_{0.20}Zr_{0.20}NiSn$**: 63(2) % of total sample mass, crystallite size: d = 40(3) nm; microstrain: η = 0.18(3) % (R_{Bragg} = 7.28, R_F = 5.04)

And other minor impurity phases.

R_p = 5.33, R_{wp} = 7.61, R_{exp} = 0.02

S.2. TEM and SEM micrographs

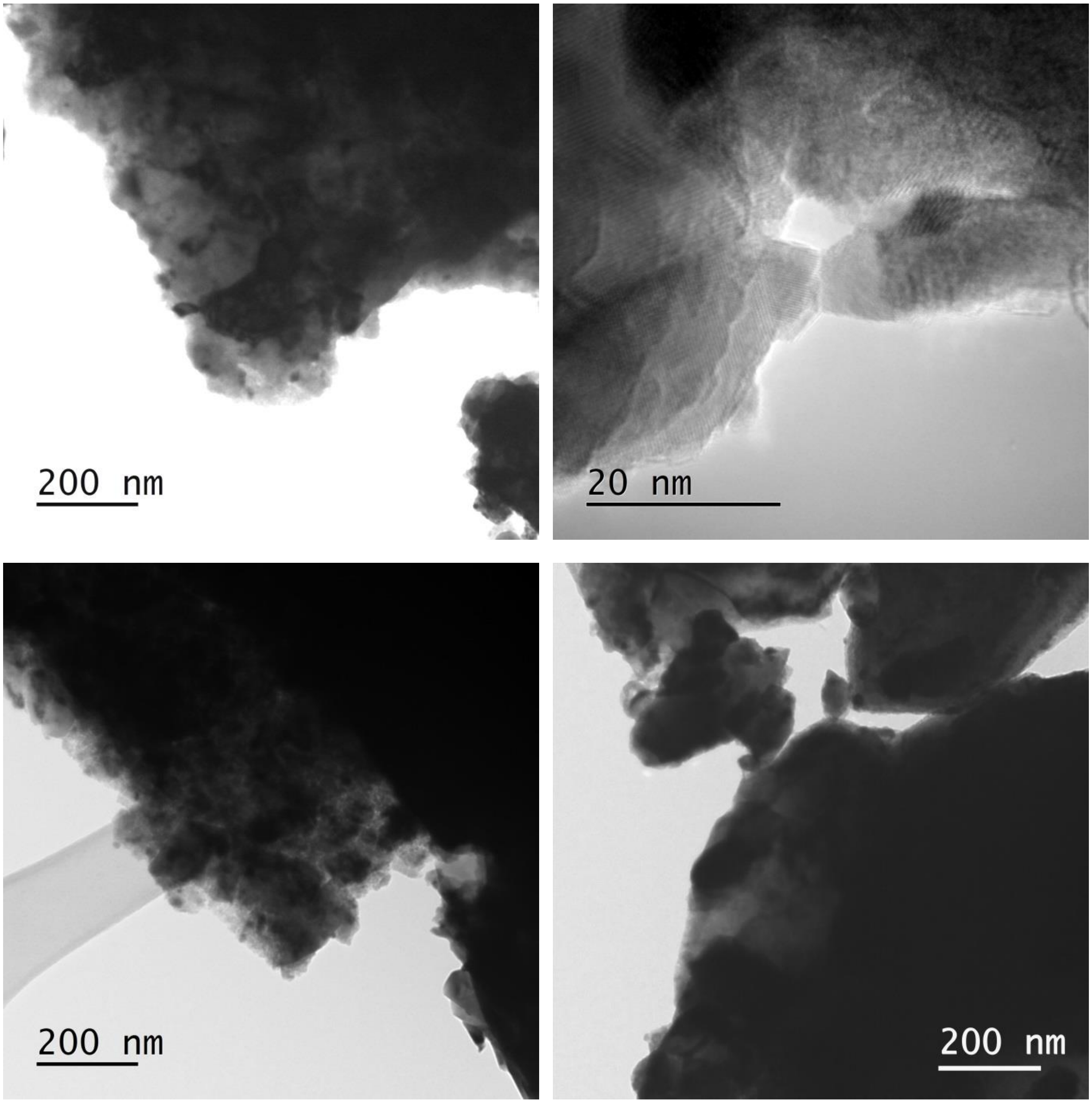

Figure S.1. TEM pictures of a ZrNiSn sample after SPS. Crystallites of varying size can be observed, in qualitative agreement with the volume-weighted average crystallite size as obtained by refinement of SR-PXD data.

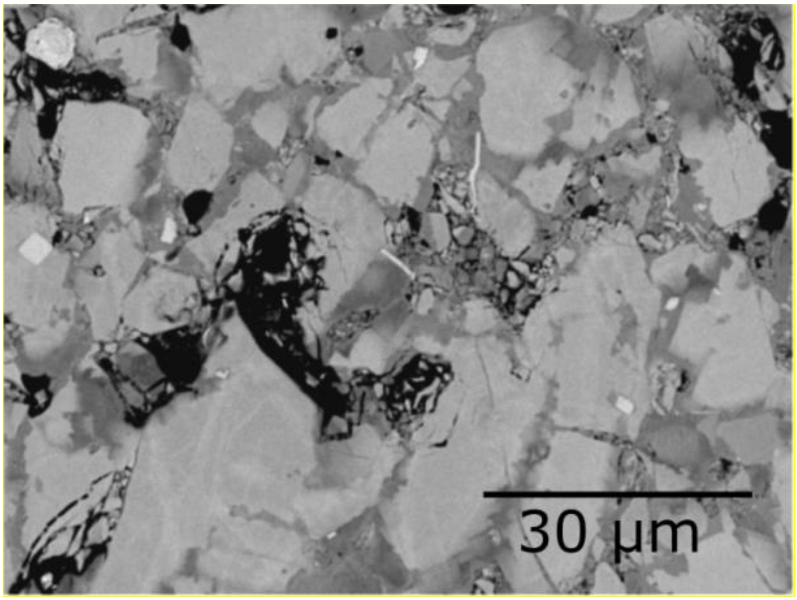

Figure S.2. SEM micrograph of the $Hf_{0.5}Ti_{0.5}NiSn$ sample. Phase contrast between two major compositions is visible. Bright areas correspond to a Hf-rich, dark areas to a Ti-rich phase. Composition as obtained by EDS agrees well with the results from PXD. The SEM images depict areas of homogeneous composition, with dimensions ranging from around 1 to 20 μm. These areas consist of several smaller crystallites below the resolution limit of the SEM used here, as illustrated by the TEM micrographs in Fig. S.1.

Composition	d_{Exp} [g/cm^3]	d_{Theo} [g/cm^3]	Relative Density
HfNiSn	10.32	10.51	98.2 %
ZrNiSn	7.30	7.80	93.5 %
TiNiSn	6.86	7.17	95.7 %
$Hf_{0.38}Zr_{0.62}NiSn$	8.29	8.85	93.7 %
$Zr_{0.5}Ti_{0.5}NiSn$	6.96	7.51	92.7 %
$Hf_{0.5}$Ti0.5NiSn	8.26	8.9	92.8 %
$Hf_{0.25}Zr_{0.25}Ti_{0.5}NiSn$	8.20	8.21	100 %

Table S.1. Experimental gravimetric density d_{Exp} compared to the theoretical density d_{Theo} for the samples investigated here.

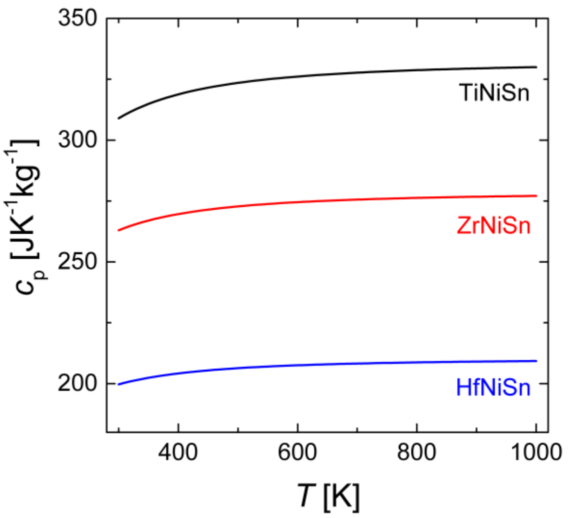

Figure S.3. Specific heat capacity c_P as a function of temperature for the three unmixed compositions. Values for the mixed compositions are obtained by interpolating the unmixed compositions.

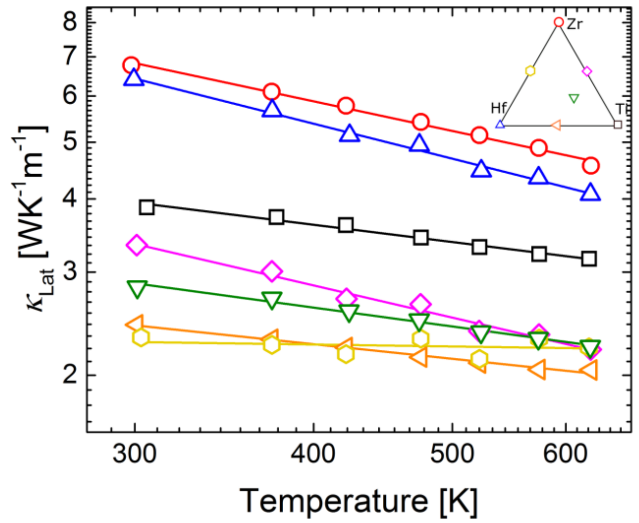

Figure S.4. Experimental κ_{Lat} as shown in Fig. 5. Solid lines represent the best fit $\kappa_{Lat} \propto T^{-x}$, to obtain the temperature exponent x.
